# Inhibitory Effect of an Acidic Peptide on the Activity of an Antimicrobial Peptide from the Scorpion *Mesobuthus martensii* Karsch

**DOI:** 10.3390/molecules23123314

**Published:** 2018-12-14

**Authors:** Wanxia Shi, Pengchen He, Xian-Chun Zeng, Weiwei Wu, Xiaoming Chen

**Affiliations:** State Key Laboratory of Biogeology and Environmental Geology & School of Environmental Studies, China University of Geosciences (Wuhan), Wuhan 430074, China; shiwxia@sina.com (W.S.); Hepengchencug@163.com (P.H.); wuweiweicug@163.com (W.W.); chenxiaomingcug@163.com (X.C.)

**Keywords:** venom, highly acidic peptide, scorpion, *Mesobuthus martensii* Karsch, antimicrobial peptide

## Abstract

Highly acidic peptides with no disulfide bridges are widely present in the scorpion venoms; however, none of them has been functionally characterized so far. Here, we cloned the full-length cDNA of a short-chain highly acidic peptide (referred to as HAP-1) from a cDNA library made from the venom glands of the Chinese scorpion *Mesobuthus martensii* Karsch. HAP-1 contains 19 amino acid residues with a predicted IP value of 4.25. Acidic amino residues account for 33.3% of the total residues in the molecule of HAP-1. HAP-1 shows 76–98% identities to some scorpion venom peptides that have not yet been functionally characterized. Secondary structure prediction showed that HAP-1 contains a beta-sheet region (residues 9–17), and two coiled coil regions (residues 1–8 and 18–19) located at the N-terminal and C-terminal regions of the peptide, respectively. Antimicrobial assay showed that HAP-1 does not have any effect on the growth of the bacterium *Staphylococcus aureus* AB94004. However, it potently inhibits the antimicrobial activity of a 13-mer peptide from *M. martensii* Karsch against *Staphylococcus aureus* AB94004. This finding is the first characterization of the function of such highly acidic peptides from scorpions.

## 1. Introduction

Scorpion venom is a really rich source of bioactive peptides. Over the past decades, approximately 700 venom peptides have been identified from 56 species of scorpions [1]. These peptides include the toxins with three–four disulfide bridges, which target Na^+^, K^+^, Cl^−^, and Ca^2+^ channels, and the venom peptides without disulfide bridges, which possess antimicrobial, anti-virus, bradykinin-potentiating, or immune-regulatory activities [2,3,4,5,6,7,8,9,10,11,12,13,14,15,16,17]. The scorpion toxins characterized so far contain four different core topologies [18]. The most common core topology is cystine-stabilized α/β (CS α/β) motif in which an α-helix and a β-sheet are covalently connected by two or multiple disulfide bonds [19,20,21]. The second topology is cystine-stabilized α-helix-loop helix (CS α/α), where two short α-helices are connected by a β-turn [22]. The third fold found in scorpion-venom peptides is the inhibitor cystine knot (ICK) motif, and the fourth is disulfide-directed hairpin (DDH) motif, which is regarded as the ancestral fold of ICK [18,23]. The scorpion venom peptides without disulfide bridges display diverse primary structures, which are α-helical and cationic with 13–47 amino acid residues or are highly acidic with 19–50 residues [5,9,20]. Scorpion peptides have great potential applications in the pharmaceutical and agrochemical fields [6,9,24,25,26,27,28].

Acidic peptides are a new class of scorpion venom peptides, which have been rarely identified and poorly characterized so far. Over the past 10 years, 19 such peptides have been identified from scorpions, including BmKa1 and BmKa2 from *M. martensii* Karsch [25], SJE098.1 and SJE098.2 from *Scorpiops jendeki* [29], Q5G8B2, Q5G8A9, Q5G8B1, and Q5G8B0 from *Tityus costatus* [30], MeVP-8, AMX81473.1, ABR21055.1, ABR20121.1, and Mevtoxinlip-4 from *Mesobuthus eupeus*, ACJ23161.1 and BoiTx776 from *Buthus occitanus Israelis* [31], ADY62660.1 and HjVP from *Hottentotta judaicus* [32], and ALX72366.1 and ALY87545.1 from *Odontobuthus doriae*. The common properties of these peptides are: (i) Highly acidic with a calculated isoelectric point (IP) lower than 5.0; (ii) highly hydrophilic; (iii) composed of random coil regions and with some α-helical domains; and (iv) all these peptides have not yet been functionally characterized.

Acidic peptides are widely present in the venom glands of all detected species of scorpions. For example, the transcripts of acidic peptides are the seven^th^ most abundant accounting for 2.60% of the total venom peptide transcriptome of scorpion *Androctonus bicolor* [17]. It is therefore interesting to determine: what functions or activities do these peptides possess? In this study, we identified an acidic peptide (referred to as HAP-1) from scorpion *M. martensii* Karsch. We, for the first time, found that HAP-1 has significant inhibitory effect on the activity of a 13-mer antimicrobial peptide from the scorpion. The finding of this study may help to explain why the pore-forming activities of antimicrobial peptides in the storage cells of scorpion venom never damage the storage cells, and deepen our understandings of the pharmacological activities of scorpion venom peptides.

## 2. Results

### 2.1. Screening of the cDNA Library Made from the Venom Glands of M. martensii Karsch

From 1.0 μg of cDNA, we obtained a total of 5.56 × 10^5^ clones. This suggests that our cDNA library covers approximately 99.99% of the total transcripts generated in scorpion venom glands. We randomly picked 20 bacterial colonies from the library for quality evaluation. Plasmid DNAs extracted from these bacteria were digested with *Not* I and *Sal* I. Electrophoresis analysis for the digested products showed that among the 20 clones, 19 contain inserts of 200 bp to 500 bp, and one is the empty vector. It is well known that the precursors of scorpion venom peptides are 50–96 amino acid residues long. It can thus be inferred by the full-length cDNAs encoding these peptides should be 250–600 bp long. Therefore, the cDNA inserts of our cDNA library represent nearly intact mRNA pools coding for scorpion venom peptides.

**Figure 1:**
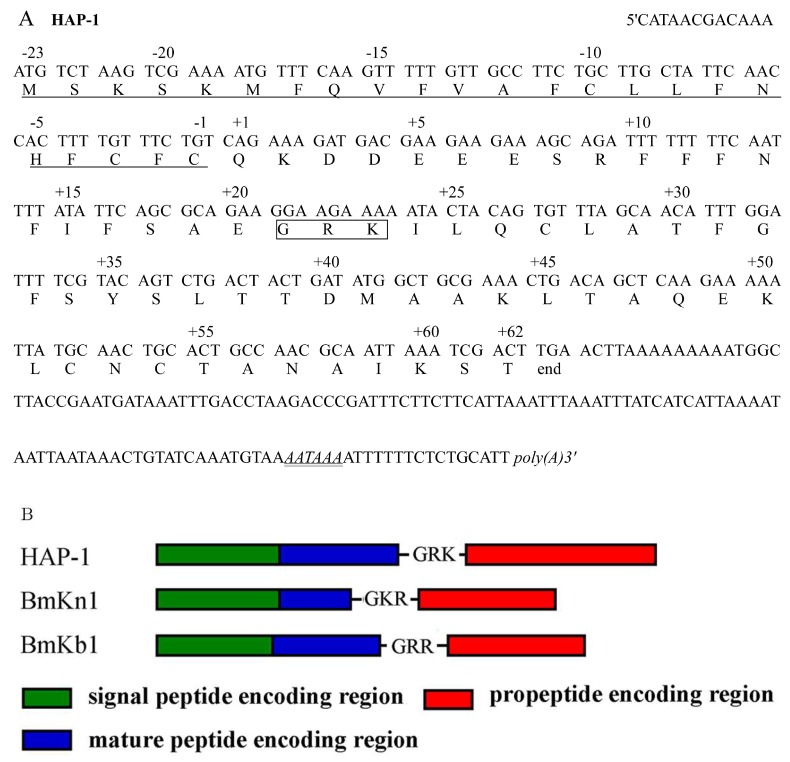
Precursor of HAP-1. (**A**) The cDNA and protein sequences of HAP-1. The deduced amino acid residues are given below the corresponding nucleotide codons. The amino acid residues sequence is numbered starting from the N-terminal residue of the peptide; the first residue of the mature peptide is numbered as +1, whereas the signal peptide is numbered as minus. The signal peptide is underlined; a putative polyadenylation signal (*AATAAA*) is marked by italic; the posttranslational processing signal is marked with a box. (**B**) Comparison of the precursor structure of HAP-1 with those of some antimicrobial peptides from the scorpion *M. martensii* Karsch.

**Figure 2:**
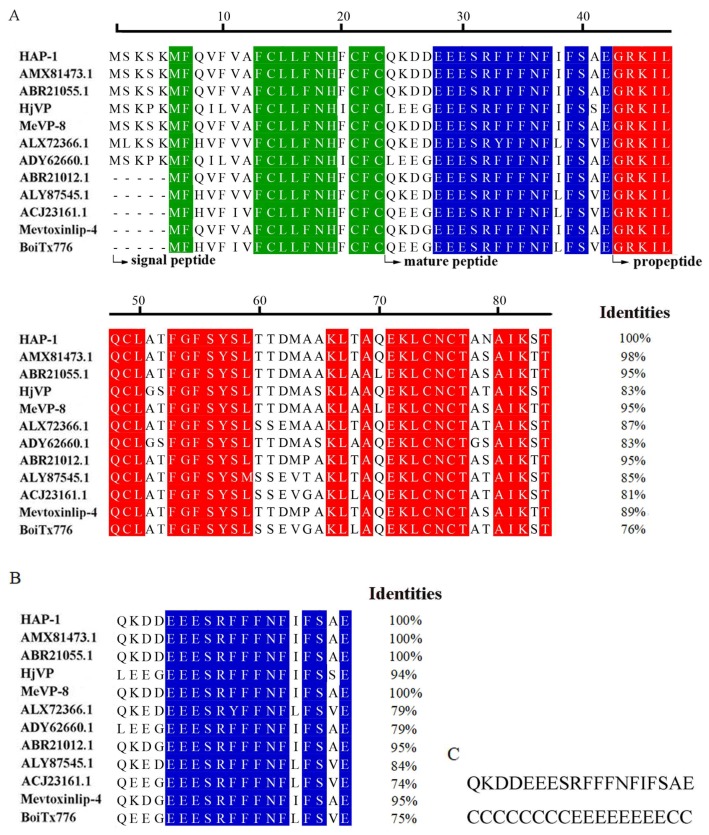
Bioinformatic characterization of HAP-1. (**A**) Multiple sequence alignment of the precursors of HAP-1 and its homologues. The signal peptides, matured peptides, and propeptides are highlighted in green, blue, and red colors, respectively. (**B**) Multiple sequence alignment of the mature peptides of HAP-1 and its homologues. (**C**) Secondary structure prediction of HAP-1. C and E stand for coiled coil and β-sheet structures, respectively.

In situ screening of the cDNA library using DNA hybridization with the probe mixture showed that the majority of the bacterial colonies are positive clones; this suggests that there are very few new types of scorpion venom peptides that have not yet been identified from *M. martensii* Karsch. Screening of 3000 clones yielded 52 clones with a negative hybridization signal. DNA sequencing of these clones showed that we obtained a novel cDNA clone encoding a short-chain highly acidic peptide (referred to as HAP-1) with no disulfide-bridges, which represents a new class of scorpion venom peptides (Figure 1 and Figure 2).

### 2.2. Precursor of HAP-1

The cDNA shown in Figure 1A is 402 bp long. It contains a 5′-untranslated region of 12 bp and a 3′-untranslated region of 135 bp. A potential polyadenylation signal (*AATAAA*) is located at the positions 381-386 of the nucleotide sequence.

The precursor of HAP-1 (AGV98852.1) deduced from the cDNA consists of 84 amino acid residues. The N-terminal region has a hydrophobic signal peptide of 23 residues for endoplasmic-reticulum membrane translocation, which would be cleaved off at the residue Gln. A typical pro-peptide cleavage signal Gly-Arg-Lys was found at the sites of the residues 43–45, and the processing reactions would follow a common rule that starts with cleavage of the pro-peptide at the site of paired basic residues Arg-Lys. This reaction generates an intermediate peptide ended with Gly-COOH, which would be modified by amidating enzyme. Therefore, the processing reactions lead to formation of a mature peptide of 19 amino acid residues with an amidated C-terminal end. As shown in Figure 1B, the structural organization and processing signal of the HAP-1 precursor are similar to those of the precursors of some antimicrobial peptides from the scorpion *M. martensii* Karsch, such as BmKn1 and BmKb1 [26]. This suggests that the genes encoding HAP-1 and the antimicrobial peptides may have originated from a common ancestor.

### 2.3. Bioinformatic Characterization of HAP-1

The predicted molecular weight of HAP-1 is 3250 Da. It is a highly acidic peptide, of which predicted IP is 4.25. Protein sequence homology search indicated that the precursor of HAP-1 shows 98%, 95%, 95%, 95%, 89%, 87%, 85%, 83%, 83%, 81%, and 76% identities to those of AMX81473.1, MeVP-8, ABR21055.1, ABR20121.1, Mevtoxinlip-4, ALX72366.1, ALY87545.1, HjVP, ADY62660.1, ACJ23161.1, and BoiTx776 from scorpions, respectively (Figure 2A); the mature peptide of HAP-1 displays 100%, 100%, 95%, 100%, 95%, 79%, 84%, 94%, 79%, 74%, and 75% identities to those of the eleven peptides, respectively (Figure 2B). Except for the eleven peptides, HAP-1 shows no significant homology with any other known venom peptides from scorpions and other animals. This suggests that HAP-1, together with AMX81473.1, MeVP-8, ABR21055.1, ABR20121.1, Mevtoxinlip-4, ALX72366.1, ALY87545.1, HjVP, ADY62660.1, ACJ23161.1, and BoiTx776 represents a novel class of scorpion venom peptides.

Secondary structure prediction showed that HAP-1 is composed of two coiled coil regions (residues 1–8 and 18–19), which are separated by a β-sheet strand (residues 9–17) (Figure 2C). In comparison, the circular dichroism (CD) spectroscopy analysis indicated that the secondary structure of HAP-1 consists of 27% helix, 28% beta-sheet, 19% beta-turn, and 26% random coil, as determined in 50 mM phosphate buffer saline (Figure S1). Such structure significantly differs from those of other acidic peptides from scorpions, which mainly consist of coiled coil domains [26].

### 2.4. HAP-1 Markedly Inhibits the Activity of an Antimicrobial Peptide from M. martenssi Karsch

To determine the function of HAP-1, we tested whether it has antimicrobial activity or not. Antimicrobial assay was performed against some representative species of Gram-positive bacteria, including *Staphylococcus aureus* AB94004, *Bacillus magaterium* AB90008, *Bacillus thuringiensis* AB93100, and Gram-negative bacteria, including *Escherichia coli* DH5α, *Escherichia coli* JM109, *Pseudomonas putida*, *Pseudomonas fluorescens*, *Klebsiella oxytoca* AB2010143, *Enterobacter cloacae* AB2010162, *Salmonella enterica* AB2010185, as well as fungi *Candida tropicalis* AY91009, as described previously [5], using the synthetic HAP-1 peptide at a maximal concentration of 80 μM. Unfortunately, the result showed that HAP-1 has no significant antimicrobial activity.

We further tested whether HAP-1 possesses synergistic effect on the antimicrobial activity of ApHt_20 (GFWGSLWEGVKSVI), an IsCT-like venom peptide from *M. martensii* Karsch. The result showed that HAP-1 has no synergistic effect on the antimicrobial activity of ApHt_20.

**Figure 3:**
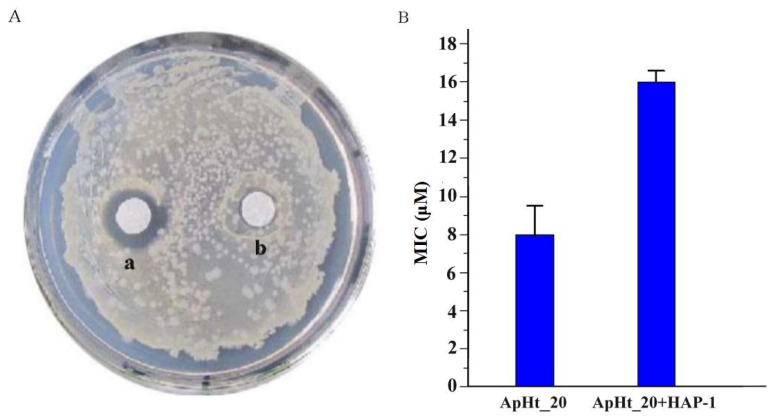
The inhibitory effect of HAP-1 on the antimicrobial activity of ApHt_20 (GFWGSLWEGVKSVI) against *Staphylococcus aureus* AB94004. (**A**) The agar diffusion assay. Disc a contains 40 nM of ApHt_20; Disc b contains 40 nM of ApHt_20 and 140 nM of HAP-1. (**B**) MICs of ApHt_20 against *Staphylococcus aureus* AB94004 in the presence/absence of 32 μM HAP-1.

It is interesting to see that HAP-1 is able to markedly inhibit the activity of ApHt_20 against *Staphylococcus aureus* AB94004 (Figure 3). As shown in Figure 3A, we performed an antimicrobial assay by placing sterile paper discs containing different concentrations of ApHt_20 onto the plate, which was coated by bacterial cells. The results showed that when 40 nM or higher concentrations of ApHt_20 were added, the inhibition zone around disc was completely transparent; however, higher concentrations of ApHt_20 cannot increase the size of the inhibition zone due to limited diffusibility of peptides in the agar. Thus, we used 40 nM of ApHt_20 to perform the antimicrobial assay in the presence of HAP-1. We found that the minimum concentration of HAP-1 required to completely inhibit the generation of the inhibition zone is 140 nM.

As shown in Figure 3B, ApHt_20 inhibits the growth of *Staphylococcus aureus* AB94004 with the minimal inhibitory concentration (MIC) of 8 μM; however, if 32 μM of HAP-1 was added to the culture, the MIC of ApHt_20 increased to approximately 16 μM. As a negative control, the heated HAP-1 peptide (treated at 120 °C for 30.0 min) failed to block the antimicrobial activity of ApHt_20.

It was shown that the pH value of the medium is a factor that significantly affects the MIC of an antimicrobial peptide against certain bacterium. We thus analyzed the pH value of the medium. We found that the presence of 32 μM HAP-1 had no detectable effect on the pH value of this medium. This observation excluded the possibility that the inhibitory effect of HAP-1 on the antimicrobial activity of ApHt_20 was attributed to that this acidic peptide could reduce the pH level of the cultural medium; more likely, the inhibition was resulted from the molecular interaction between HAP-1 and ApHt_20.

Therefore, HAP-1 markedly inhibits the antimicrobial activity of ApHt_20 from *M. martensii* Karsch.

## 3. Discussion

*M. martenssi* Karsch is a representative scorpion species in Asia. It has been used as a key traditional medicine to relieve pain and treat some other diseases such as epilepsy, tetanus, rheumatism, stoke, and tumors for thousands of years [33,34]. Thus, it is fascinating to identify and functionally characterize pharmacologically active components from the venom of the scorpion, and gain new insights into the molecular mechanisms of actions. We have focused on the identification and functional characterization of new venom peptides from this scorpion since 1999 [14]. Until now, at least 120 different venom peptides have been identified from *M. martensii* Karsch by our group and other researchers (see GenBank database).

Acidic peptides are a new group of venom peptides from scorpions, which can be classified into two subfamilies: One includes BmKa1, SJE098.1, SJE098.2, Q5G8B2, Q5G8A9, Q5G8B1, Q5G8B0, and BmKa2, and the other includes MeVP-8, Mevtoxinlip-4, HjVP, BoiTx776, AMX81473.1, ABR21055.1, ABR20121.1, ALX72366.1, ALY87545.1, ADY62660.1, ACJ23161.1, and HAP-1 [25,29,30,31,32]. However, none of these acidic peptides have been functionally characterized so far. As these peptides display no significant homology with any other peptides that were functionally described before, it is tough to predict their functions. Here, we found that HAP-1 possesses no detectable antimicrobial and hemolytic activity when 80 μM of HAP-1 was added to the bacterial culture. However, HAP-1 is able to potently inhibit the activity of a 13-mer antimicrobial peptide (ApHt_20) from *M. martensii* Karsch. This suggests that the highly acidic peptides may function as a regulator of the activities of the antimicrobial peptides from the scorpion venom. The finding of this work has deepened our understandings of scorpion venom peptides, and may explain why the pore-forming activities of antimicrobial peptides in the storage cells of scorpion never damage the storage cells themselves.

HAP-1 is a highly acidic peptide while ApHt_20 is positively charged. However, we found that HAP-1 is not able to change the pH level of the medium at a concentration as high as 80 μM. This suggests that the inhibitory effect of HAP-1 on the antimicrobial activity of ApHt_20 could be attributed to the direct interaction of the two molecules, instead of the pH value changes of the medium. This interaction would change the alpha-helix structure of ApHt_20 that is required for its antimicrobial activity.

## 4. Materials and Methods

### 4.1. Scorpion Treatment and Purification of Total mRNA from the Venom Glands

The frozen telsons of the scorpion *M. martensii* Karsch were gifted from Prof. Wenxin Li, College of Life Sciences, Wuhan University, China. All the operations of animal treatment were approved by the Institutional Animal Care and Use Committee of Wuhan University (No. ECCLS20120076), and were conducted as described previously [35]. The glands were thoroughly ground in a mortar with a pestle in the presence of liquid nitrogen. Total RNA was extracted from the homogenized venom glands using RNAiso Plus. Poly(A)^+^ mRNA was purified using Oligotex^TM^-dT30 mRNA purification Kit (TaKaRa, Dalian, China).

### 4.2. Construction of a cDNA Library

A cDNA library of the scorpion venom glands was constructed using the TaKaRa cDNA Library Construction kit as described by the manufacturer. Briefly, first and double-stranded cDNA was synthesized from 2.0 mg of the purified mRNA using M-MLV Reverse Transcriptase and Oligo(dT)18 Anchor Primer (Invitrogen, Carlsbad, CA, USA). After that, synthesized cDNA was exacted with phenol-chloroform-isoamyl, and was precipitated with ethanol. Purified cDNA was then ligated into *EcoR* I-*Sma* I adaptor, and digested with *Not* I. After removal of excess adaptors and the short-chain cDNA using a Spin column, the cDNA was ligated into pAP3neo Predigested Vector. Ligated cDNA was introduced into the competent *Escherichia coli* DH5α cells by transformation.

### 4.3. Screening of the cDNA Library

*M. martensii* Karsch is well characterized in its venom peptides. At least 100 different peptides have been identified from this scorpion. Therefore, it is essential to remove redundant or highly expressed known cDNAs when a random sequencing strategy was used to isolate new cDNA clones. In situ screening of bacterial colonies was performed with DNA probe hybridization. The DNA probe is a mixture of cDNA fragments including the coding sequences of BmKbpp, BmKCT, BmKBT, BmKT, BmKK2, BmTx1, and BmM4. The probes were ^32^P-labeled using PCR strategy with a mixture of primers corresponding to all the probe cDNAs. Individual colonies are replicated from the plate to a nylon hybridization filter. The filter was placed on an OmniTray previously filled with LB agar containing the Kanamycin antibiotic, and incubated overnight at 37 °C, allowing the cells to grow on the filter. The bacteria were lysed with alkaline, and the filter was neutralized using 1.0 mL of 1.0 M Tris (pH 5.4). After being air-dried, the filter was hybridized with ^32^P-labeled probes. Auto-radiogram was thus developed after exposure with intensifying screen at −80 °C for 5 h. We selected the clones with negative signals and with the inserts ranging from 250 bp to 500 bp for sequencing analysis.

### 4.4. Bioinformatics Analysis

Amino acid residues sequence homology searches were performed using the BLAST software (http://www.ncbi.nlm.nih.gov/BLAST). Physico-chemical property of a peptide was predicted by the ProtParam tool (http://expasy.org/tools/protparam). Protein secondary structure was predicted using the YASPIN program (http://www.ibi.vu.nl/programs/yaspin). Putative signal peptide cleavage site was determined using the SignalP 3.0 server (http://www.cbs.dtu.dk/services/SignalP/).

### 4.5. Bacterial Plaque Inhibition Assay

Bacteria were grown in the TSB (Tryptic Soy Broth) medium into an OD_600_ of 0.6. About 0.1 mL of the culture containing approximate 1 × 10^8^ cells was spread over the TSB agar plate. A small sterile paper disc (5 mm) containing peptides was placed onto the plate. The dishes were incubated overnight at 37 °C. The dimension of the bacterial growth inhibition zone was thus measured. Minimum inhibitory concentration (MIC) of the antimicrobial peptide was also determined, as previously described [5].

## 5. Conclusions

In this study, we cloned the full-length cDNA of a short-chain highly-acidic peptide (referred to as HAP-1) from a cDNA library made from the venom glands of the Chinese scorpion *Mesobuthus martensii* Karsch. Using bioinformatical analysis, we found that HAP-1 contains 19 amino acid residues with the predicted IP value of 4.25. Acidic amino residues account for 33.3% of the total residues in the molecule of HAP-1. Secondary structure prediction showed that HAP-1 contains a beta-sheet region (residues 9–17), and two coiled coil regions (residues 1–8 and 18–19) located at the N-terminal and C-terminal regions of the peptide, respectively. HAP-1 possesses no detectable antimicrobial and hemolytic activity when 80 μM of HAP-1 was included in the bacterial culture. Antimicrobial assay showed that HAP-1 does not have any effect on the growth of the bacterium *Staphylococcus aureus* AB94004. However, it potently inhibits the antimicrobial activity of a 13-mer peptide from *M. martensii* Karsch against *Staphylococcus aureus* AB94004. This finding is the first characterization of the function of the highly acidic peptides from scorpions. This work may help to fully understand the physiological functions of scorpion venom, and gains new insight into the mechanism by which the pore-forming activities of antimicrobial peptides in the venom storage cells of scorpions never damage the storage cells.

## Supplementary Materials

Supplementary materials are available online.
