# Supplementary material for: Inhibitory Effect of an Acidic Peptide on the Activity of an Antimicrobial Peptide from the Scorpion Mesobuthus martensii Karsch
Source: Molecules. 2018 Dec 14;23(12):3314. doi: 10.3390/molecules23123314 (PMC6321396; doi:10.3390/molecules23123314)
Supplement: Supplementary file 1 [file molecules-23-03314-s001.pdf]

Supplementary

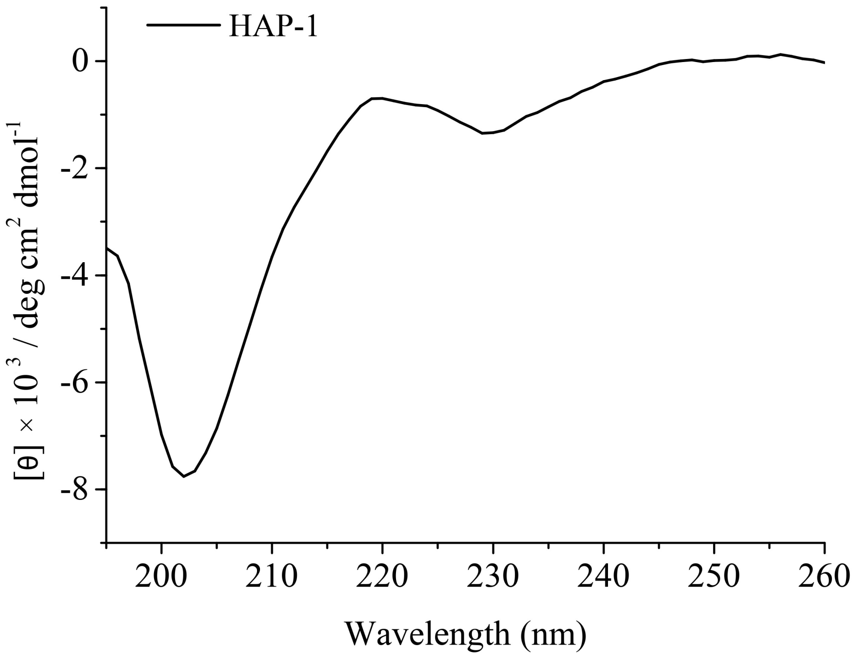

Figure S1. Far-UV CD spectrum of HAP-1. The concentration of the peptide was 1.0 mg/mL. Temperature was 25 ℃.

Table S1. Deconvoluted CD spectra of HAP-1.

**Peptide**	**helix**	**β-sheet**	**β-turn**	**random coil**
HAP-1	27%	28%	19%	26%
